# A probabilistic algorithm to process geolocation data

**DOI:** 10.1186/s40462-016-0091-8

**Published:** 2016-11-18

**Authors:** Benjamin Merkel, Richard A. Phillips, Sébastien Descamps, Nigel G. Yoccoz, Børge Moe, Hallvard Strøm

**Affiliations:** 1 Norwegian Polar Institute, Fram Centre, P.O. Box 6606, Langnes, NO-9296 Tromsø, Norway; 2 Department of Arctic and Marine Biology, UiT The Arctic University of Norway, NO-9037 Tromsø, Norway; 3 British Antarctic Survey, Natural Environment Research Council, High Cross, Madingley Road, Cambridge, CB3 0ET UK; 4 Norwegian Institute for Nature Research, P.O. Box 5685, Sluppen, NO-7485 Trondheim, Norway

**Keywords:** Animal tracking, Global Location Sensors, GLS, Method assessment, Sea surface temperature, Probability sampling, probGLS, Threshold method

## Abstract

**Background:**

The use of light level loggers (geolocators) to understand movements and distributions in terrestrial and marine vertebrates, particularly during the non-breeding period, has increased dramatically in recent years. However, inferring positions from light data is not straightforward, often relies on assumptions that are difficult to test, or includes an element of subjectivity.

**Results:**

We present an intuitive framework to compute locations from twilight events collected by geolocators from different manufacturers. The procedure uses an iterative forward step selection, weighting each possible position using a set of parameters that can be specifically selected for each analysis.

The approach was tested on data from two wide-ranging seabird species - black-browed albatross *Thalassarche melanophris* and wandering albatross *Diomedea exulans* – tracked at Bird Island, South Georgia, during the two most contrasting periods of the year in terms of light regimes (solstice and equinox). Using additional information on travel speed, sea surface temperature and land avoidance, our approach was considerably more accurate than the traditional threshold method (errors reduced to medians of 185 km and 145 km for solstice and equinox periods, respectively).

**Conclusions:**

The algorithm computes stable results with uncertainty estimates, including around the equinoxes, and does not require calibration of solar angles. Accuracy can be increased by assimilating information on travel speed and behaviour, as well as environmental data. This framework is available through the open source R package probGLS, and can be applied in a wide range of biologging studies.

**Electronic supplementary material:**

The online version of this article (doi:10.1186/s40462-016-0091-8) contains supplementary material, which is available to authorized users.

## Background

The ability to track animals across large distances in space and time has revolutionized our understanding of their movements during the breeding and nonbreeding seasons [1, 2]. Thanks to the development of light-level data loggers (geolocators; also termed Global Location Sensor or GLS loggers) [3], we are now able to track small animals which cannot carry heavy satellite-transmitters or GPS (‘global positioning system’) loggers (e.g. [4, 5]). Indeed, geolocators are used very frequently on nonbreeding seabirds, because long-term deployment of satellite or GPS devices using harnesses is a major welfare concern (e.g. [6]) and also on other marine organisms, including fish, that rarely, if ever, are at the sea surface and so cannot be tracked using radio wave technology. Currently, miniaturized GPS loggers in the same weight range as geolocators record few locations throughout the deployment period; thus, the data are unsuitable for answering ecological questions on finer temporal scales.

Geolocators record ambient light intensities and elapsed time, from which longitude and latitude can be estimated [3, 7]. They can record data for up to a year or longer, and cover one or several annual migration cycles [8, 9]. Their small size and mass (to <1 g) allow a wide range of species to be tracked, and because of the relatively low cost (compared with miniaturized GPS devices), they can be used to track many individuals for multi-population studies (e.g. [10–13]).

A number of methods have been developed to estimate locations from light data (Table 1), and to filter the resulting outputs in various ways [14–17]. These are mainly based on either a threshold [7, 18] or template-fit approach [19]. In the former, longitude is computed from the timing of local noon, and latitude from day length, based on the timing of twilight events (i.e. dusk and dawn) which are determined using a pre-defined light intensity threshold. Further, latitude depends on the solar angle below the horizon at which the threshold is crossed [7]. This sun elevation angle, which is affected by shading during the twilight period (related to behaviour and activity patterns as well as weather), and latitude [20], has to be calibrated, and for practical purposes, is generally assumed to stay constant during the entire deployment period. In contrast, the template-fit method involves fitting a simplified geophysical model for various latitudes (i.e. the template) to recorded light intensities for each day at a longitude estimated in the same way as in the threshold method [21].

**Table 1:** Comparison of available methods to process geolocation data

	Hill 1994 [7], Hill & Braun 2001 [18]	Teo et al. 2004 [32]	Domeier et al. 2005 [33]	Royer et al. 2005 [34]	Ekstrom 2007 [21]	Nielsen et al. 2006 [24], Lam et al. 2008 [35]	Tremblay et al. 2009 [28]	Sumner et al. 2009 [26]	Nielsen & Sibert 2007 [25], Lam et al. 2010 [36]	Rakhimberdiev et al. 2015 [27]	this study
Principle to infer locations from light data	threshold	threshold (only longitude)	threshold (only longitude)	-	template fit	threshold	-	curve model	template fit	template fit	threshold
Data needed for method	twilight events	twilight events	twilight events	“raw” locations	full light range data	twilight events	“raw” locations	clipped light range data	full light range data	clipped light range data	twilight events
R package	GeoLight [23]					Ukfsst		SGAT, Trip Estimation	Trackit	FlightR	probGLS
Account for difference in shading					+			+		+	+
Account for movement between twilight events								+	+	+	
Estimated locations during equinox		+	+		+	+		+	+	+	+
Uncertainty estimates				+	+	+	+	+	+	+	+
Spatial error structure			constant	constant	estimated through the geolocation process	ad hoc parametric model	constant	estimated through the geolocation process	estimated through the geolocation process	estimated through the geolocation process	estimated through the geolocation process
State space model				+		+		+	+	+	
Optimisation		best match for latitude	least cost track	particle filter	least squares	unscented Kalman filter	probability sampling	MCMC (block update)	unscented Kalman filter	particle filter	probability sampling
Land scape mask		+		+			+	+		+	+
Optional/ mandatory environmental characteristics		/SST	/SST	/SST, depth		SST, depth/	SST/	SST/	SST, depth/	possible to implement/	SST, depth, sea ice …/
Optional/ mandatory speed input		+/	/+	/+				/+		+/	+/
Developed mainly for	all organisms	fish	fish	fish	all organisms	fish	marine organisms	marine organisms	fish	terrestrial birds	marine organisms

Unlike other tracking methods, locations derived from light data lack a constant spatial error structure. Latitudes are most accurate (i.e. least affected by shading) where the timing of twilight events is most distinct, i.e., during solstices and at high latitudes [7]. However, within the Arctic or Antarctic circles, position estimates are impossible around the solstices due to the lack of twilight events (i.e. polar night and midnight sun). In contrast, the error in latitude (due to shading) is highest during the equinoxes where day length is the same around the globe, and around the equator where there is little variation in day length [7].

Given the wide range of alternative methods and potential observer-specific biases, there would clearly be advantages in determining a common method for analysing all geolocation data. Any method that requires raw light values and not just timing of twilight events (Table 1) cannot be applied to data from all brands of geolocators. For instance, Lotek geolocators (Lotek Wireless Inc., Ontario, Canada) do not store these data by default and have been deployed in many studies of marine organisms. The aim of this paper is to propose an intuitive, probabilistic algorithm, implemented in R [22] through the new package probGLS, that can be used on data from all existing geolocator brands. Our method is relatively simple, easy to implement, fast to compute (compared to other more complex methods), does not require the use of a constant solar angle (as needed in the GeoLight package [23]), provides uncertainty estimates, can incorporate additional information to increase accuracy (e.g. land avoidance for marine organisms), and greatly reduces location error around the equinoxes (if additional information is available) without making assumptions about behavioural states as in state space models (SSM, e.g. [24–27]). Here we validate the approach for two open landscape species (flying seabirds), but its usability would need to be confirmed for other organisms, particular those that dive or live in closed terrestrial habitats (e.g. forests).

## Methods

### Method principle

**Fig. 1:**
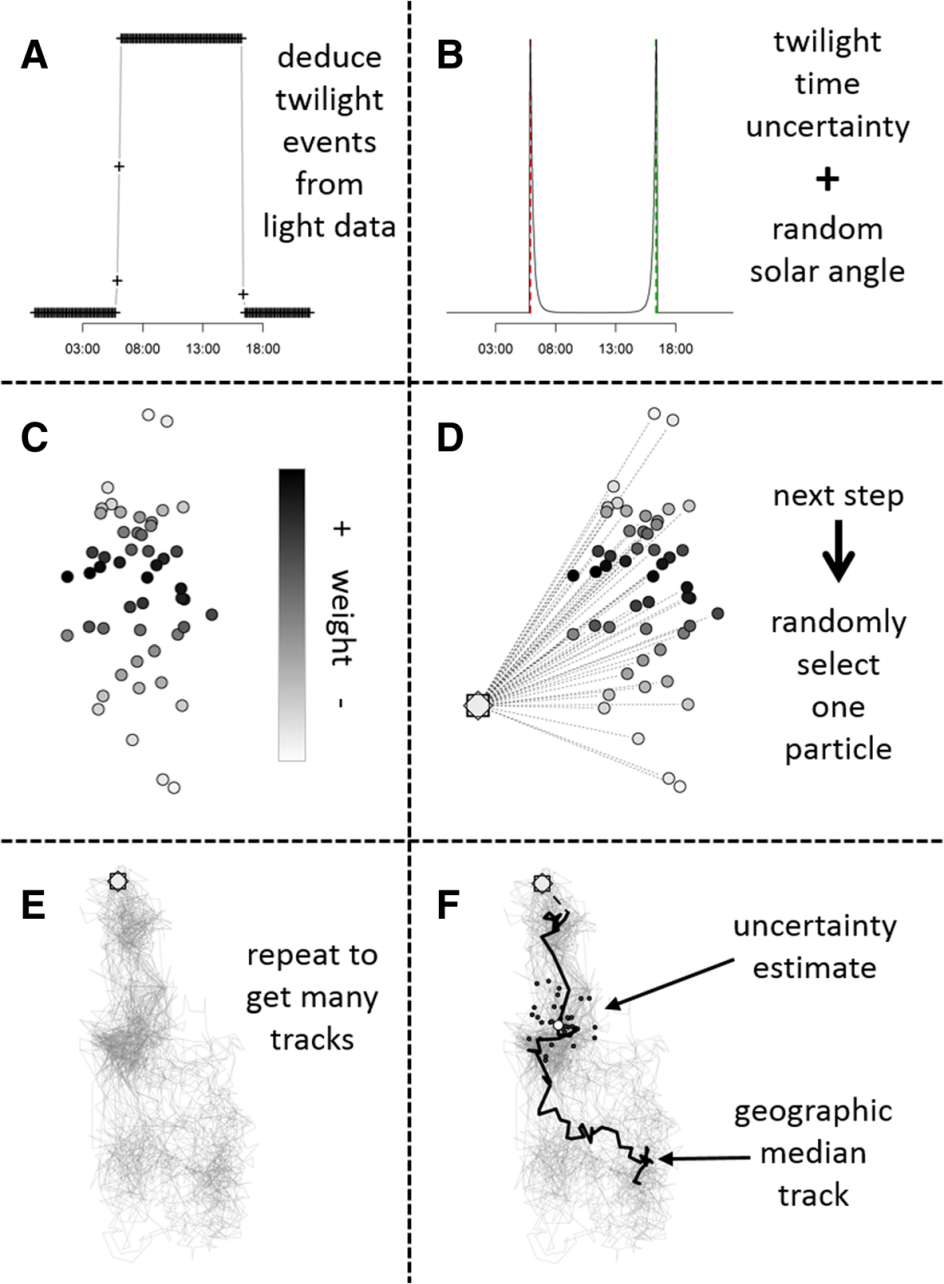
Description of the probabilistic algorithm. Timing of twilight events are either deduced from raw light data or extracted from logger specific software (**a**). Each set of twilight events is replicated by the number of particles and an uncertainty as well as a random solar angle are added to compute a cloud of possible locations (**b**). These calculated particle locations for a set of twilight events are weighted by any other chosen parameter (**c**). For each step one random particle based on their weights is chosen (**d**) and this process is repeated (**e**). The geographic median track is computed as most likely track and each modelled location has an estimated uncertainty based on all iterated tracks (**f**). This figure is modified after Figure 1 in [28]

The method is an iterative forward step selection based on [28]. The algorithm uses twilight events (Panel A, Fig. 1) identified using a range of brand-specific software for analysing light data (e.g. TransEdit2, British Antarctic Survey (BAS), Cambridge, UK), the twilightCalc function (GeoLight package; also incorporated into IntiProc, Migrate Technology, Cambridge, UK), or in the case of Lotek loggers by back-calculating twilight thresholds from computed locations as implemented in the lotek_to_dataframe function (probGLS package, this study). The framework can incorporate various sources of uncertainty (e.g. uncertainty in solar angle) as well as knowledge of the behaviour and habitat use of the study species (e.g. travel speed), by defining associated parameter values a priori (Table 3). The main steps are described below:

1. The algorithm assumes that the first position at time *t*_*1*_ is known without error (i.e. release location), regardless of the time difference between *t*_*1*_ and the first twilight event.
2. The next available pair of twilight events (dusk/dawn or dawn/dusk) is replicated x times with an additional twilight error term (from a log-normal distribution *N, μ* and *σ* on the log scale = user-defined, See Additional file 1 for information about setting these parameters) and a random solar angle (from a user-defined range) applied to each twilight before a location is calculated (Panel B, Fig. 1).
3. Using the threshold method and the twilight events computed in step 2, a cloud of positions (i.e. particles) at *t*_*i*_ is calculated. To make computations more robust, all particles outside a defined boundary box (based on known range) are removed. Further, latitudes are unreliable for a variable period around the equinoxes. For these periods (user-defined), random latitudes (with uniform distribution) within the boundary box are added to each computed longitude estimate.
4. Each particle can be weighted (i.e. given a probability of selection) according to behaviour (e.g. maximum possible speed) or environmental characteristics (e.g. sea surface temperature; Panel C, Fig. 1).
5. Then, one particle is randomly selected following a distribution based on the assigned weights (Panel D, Fig. 1). If all particles in a given cloud have a weight of 0, the entire cloud is considered unlikely and discarded.
6. The algorithm moves one time step forward to *t*_*i+1*_ and steps 2 to 5 are repeated until *t*_*n*_ (*n* being the last set of twilight events).
7. Steps 1 to 6 are iterated a set number of times to construct several probable movement paths (Panel E, Fig. 1).
8. The most likely movement path is computed as the geographic median (Additional file 2) for each computed location cloud; the variation in positions of all computed paths denotes the uncertainty at each step in time (Panel F, Fig. 1).

Tremblay et al. [28] defined their particle clouds based on “raw” locations as the geographic average with a spatial error structure. This is the case for locations derived using satellite-transmitters. However, locations estimated from light data using the threshold method can only be assumed to be the geographic average if the correct solar angle for each day is selected, shading was similar both at dawn and dusk, and the animal only moved a short distance between twilight events. If any of these conditions is violated the position could be strongly biased. Therefore, we based our method on the timing of twilight events, incorporating uncertainty and unknown solar angle (steps 2 & 3). This allows uncertainties to be incorporated that are related to differences in behaviour and weather patterns, as well as dynamic latitudinal uncertainty, which reflects the season and latitude-specific uncertainty of the geolocation method. Uncertainty in twilight events is assumed to follow a log-normal distribution. This skewed distribution takes into account that a sunrise may falsely appear to occur later, due to shading, while it is improbable that light is falsely detected prior to sunrise (and the inverse is true for sunsets). The error parameters for this uncertainty can be generated using twilight_error_estimation (package probGLS, this study). It is important that the error distribution mirrors the actual behaviour of the animal. This should be done using calibration data (i.e. ~2 weeks of data recorded on the individual at a known location). Solar angles do not have to be calibrated or assumed to be constant, but rather a reasonable range of possible angles can be defined (step 2). Also, due to the above mentioned pitfalls regarding use of “raw” locations and unknown latitude and time specific error distributions, we do not interpolate between positions to utilize the higher frequency of temperature measurements by the loggers as described by [28]. Steps 4 to 8 are in principle equivalent to [28]. However, we do not include weighted distributions of individual speeds computed using the next x particles in the record, but rather use a defined speed distribution. This is because there are no specific locations on which to base these distributions; instead, there is a cloud of possible locations. Moreover, we do not consider the geographic average track to be the most probable track, but the geographic median defined as the position with the minimum sum of all distances to all other iterated locations. Therefore the selected position will always be a computed location. In contrast, the average geographic position might, for example, be on land if the cloud of points is around a land mass, even if this is unrealistic for the study species (Additional file 2).

### Method assessment

The framework was tested using data from black-browed (*Thalassarche melanophris*) and wandering (*Diomedea exulans*) albatrosses (Table 2) tracked in December-January (incubation) and March-April (brood-guard), respectively, in 2015 from Bird Island, South Georgia (54°00’ S, 38°03’ W). All individuals were equipped with an i-gotU GPS logger (Mobile Action Technology Inc., New Taipei City, Taiwan) taped to back feathers and programmed to log a position every 10 min, and an Intigeo C250 geolocator (Migrate Technology Ltd, Cambridge, UK) attached by cable-tie to a plastic leg ring, which measured light in the range 1.1 to 74418 lux (maximum recorded at 5 min intervals) and temperature every 20 min of continuous wet (maximum, minimum and mean saved every 4 h), and tested for saltwater immersion every 6 s.

**Table 2:** Summary of tracking data available for method assessment

Species	# of individuals	# of tracks	mean ± sd (min – max) trip duration [days]	mean ± sd (min – max) # of locations per track	Deployment period
black-browed albatross	33	33	9 ± 4 (3–17)	15 ± 7 (5–31)	10 Dec 2014 to 6 Jan 2015
wandering albatross	27	32	3 ± 1 (1–7)	4 ± 2 (2–9)	14 Mar 2015 to 3 Apr 2015

Twilight events from raw light intensities were computed with twilightCalc (light threshold of 2; loggers calibrated on Bird Island). To increase precision we included sea surface temperature (SST) and land avoidance. The daily median water temperature encountered by each bird was computed from temperature data collected every 4 h by the loggers. The daily mean satellite-derived SST and mean SST error was extracted from the NOAA optimally-interpolated, high resolution SST dataset at 0.25° resolution [29]. Each movement path incorporated parameter values based on the ecology of the species and information extracted from GPS data (Table 3, and Additional file 3).

**Table 3:** Algorithm parameters used to compute locations for both assessment data sets

Model parameter	Description	Value used
particle.number	number of particles computed for each point cloud	10 000
iteration.number	number of track iterations	200
sunrise.sd & sunset.sd	shape, scale and delay values describing the assumed uncertainty structure for each twilight event following a log normal distribution	2.49/ 0.94/ 0^a^
range.solar	range of solar angles used	-7° to -1°
boundary.box	the range of longitudes and latitudes likely to be used by tracked individuals	120 W to 40 E 90 S to 0
day.around.spring.equinox & days.around.fall.equinox	number of days before and after an equinox event in which a random latitude will be assigned	includes the entire wandering albatross tracking period
speed.dry	fastest most likely speed, speed standard deviation (sd) and maximum speed allowed when the logger is not submerged in sea water	12/ 6/ 45 m/s for black-browed albatross^b^ & 12/ 7/ 70 m/s for wandering albatross^b^
speed.wet	fastest most likely speed, speed sd and maximum speed allowed when the logger is submerged in sea water	1/ 1.3/ 5 m/s^c^
sst.sd	logger-derived sea surface temperature (SST) sd	0.5 °C^d^
max.sst.diff	maximum tolerance in SST variation	3 °C
east.west.comp	compute longitudinal movement compensation for each set of twilight event [37]	used

^a^ The resulting uncertainty structure for both twilight events is illustrated in Additional file 1. These parameters are chosen as they resemble the twilight error structure of open habitat species in [20]

^b ^inferred from GPS tracks (see Additional file 3 for details)

^c^ Antarctic circumpolar current speed up to fast current speeds (i.e. Malvinas current) [38] as the tagged animal is assumed to not actively move when the logger is immerged in seawater

^d^ logger temperature accuracy

To compare GPS tracks to locations estimated from geolocator data, we calculated the average GPS location between two twilight events. Deviation for each geographic median, and nearest location (both derived from geolocator data) from the average GPS positions was computed as the great-circle distance [14]. Additionally, each average GPS position was compared to locations estimated using the classical threshold method with a fixed solar angle of -5.0° and -5.8° for black-browed and wandering albatross data, respectively. These angles give the smallest average deviation of the estimated locations from the corresponding average GPS location in a range of -1° to -7°. In addition, all positions outside the boundary box were removed (Table 2). Finally, we ran sensitivity analyses to assess how many particles (1 – 10 000) and track iterations (1 – 200) were necessary to obtain a stable and reliable track output (see R script in Additional file 4) as well as how changes in the uncertainty distribution of twilight events changes accuracy.

## Results

**Fig. 2:**
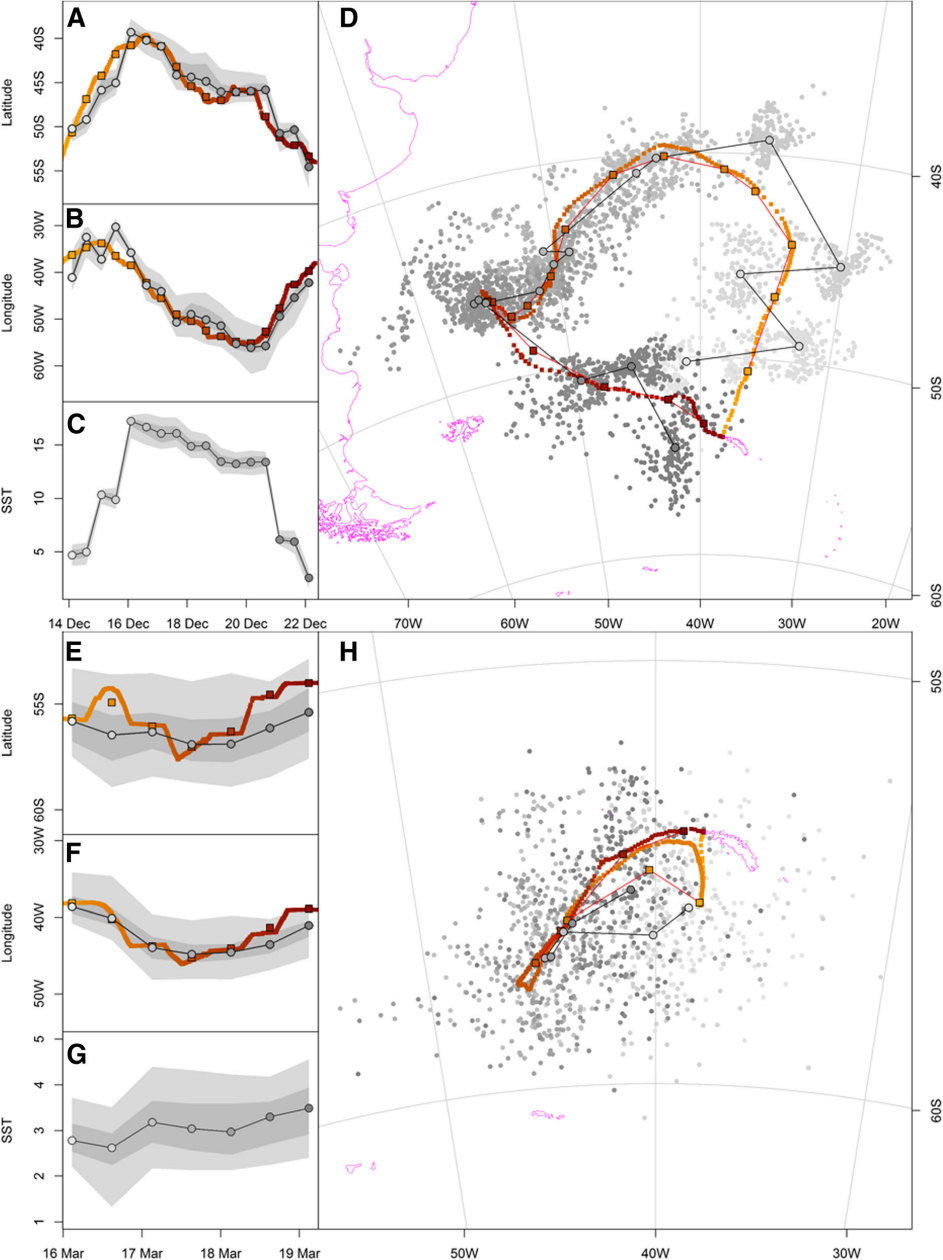
Examples trips from a black-browed albatross during the summer solstice (**a**-**d**) and a wandering albatross during the fall equinox (**e**-**h**). (**a** to **c & e** to **g**) show the change in latitude, longitude and encountered sea surface temperature (SST) with time while (**d & h**) represent the tracks. Grey scale positions show all processed geolocator locations; black framed grey positions represent median geographic geolocator locations; red symbols represent 10 min resolution GPS locations; black framed red squares are daily average GPS locations; track direction from light to dark. Shaded grey areas in (**a**) to (**c**) represents 95 and 50% uncertainty

Combined geolocator and GPS data were obtained for 33 and 27 black-browed and wandering albatrosses, respectively, in two contrasting periods characterized by minimal (solstice) and maximal (equinox) uncertainty in latitude estimation using light data (Table 2). Examples for a black-browed albatross track during the summer solstice and a wandering albatross track during the fall equinox showing both processed geolocator and GPS locations are illustrated in Fig. 2. The overall median distance between the most probable geolocator and mean GPS locations was 185 km (range 5 to 2740 km) and 145 km (range 8 to 493 km) for tracks during the summer solstice and fall equinox, respectively (Table 4, Additional file 5). The median closest distance of each iterated location cloud to the mean GPS location was 19 km and 17 km during the summer solstice and fall equinox, respectively. Using the threshold approach with a constant solar angle of -5.0° and -5.8° resulted in median distances to average GPS locations of 226 km (22% lower accuracy than the new method) and 662 km (357% lower accuracy) for the black-browed albatross data during the summer solstice and wandering albatross data during the fall equinox, respectively. Moreover, only 54% of positions could be calculated using the threshold method with the GeoLight package and a constant angle of -5.8° during the fall equinox compared to our new approach (Table 3).

**Table 4:** Summary of number of locations estimated and distance to average GPS position using two methods of light level location estimation

Species and time period	Method	# of locations	Median distance to GPS location [km]	Mean ± sd (min – max) distance to GPS location [km]
black-browed albatross during solstice	- 5.0° sun elevation	504	226	347 ± 448 (13 – 4170)
	geographic median particle	482	185	235 ± 218 (5 – 2740)
	particle cloud	482	19	66 ± 168 (0 – 2380)
wandering albatross during equinox	- 5.8° sun elevation	79	662	1225 ± 1478 (80 – 5925)
	geographic median particle	148	145	155 ± 82 (8 – 493)
	particle cloud	148	17	25 ± 24 (1 – 133)

Geographic median particle refers to the calculated most probable movement track, and particle cloud refers to the minimum distance of the iterated particle cloud from the GPS location (see Methods for details). Black-browed albatrosses were tracked around the solstice and wandering albatrosses around the equinox

**Fig. 3:**
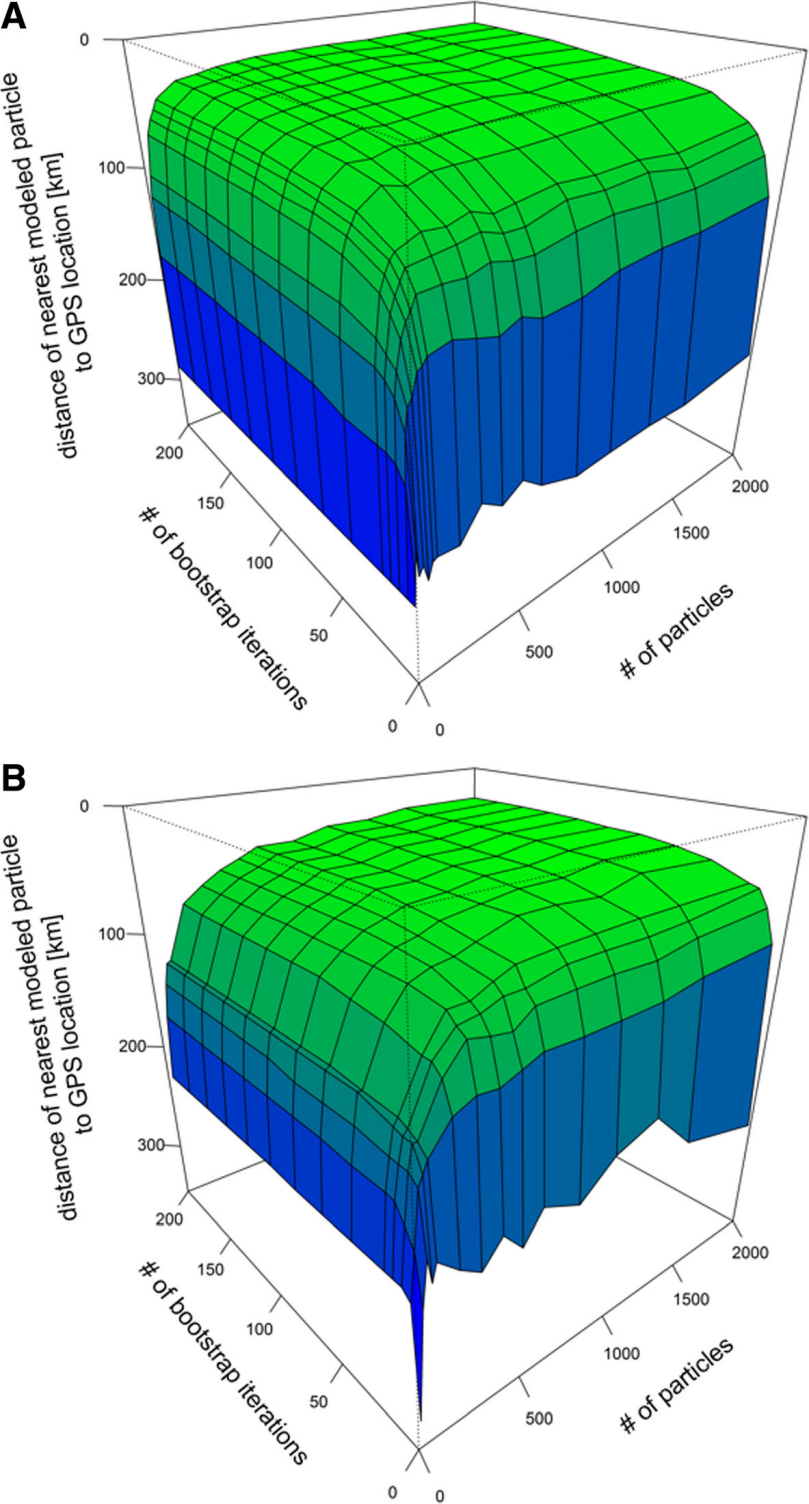
Median distance between the nearest particle and its associated average GPS location in relation to number of iterations and number of particles used. **a** Black-browed albatross data during the summer solstice; **b** Wandering albatross data during the fall equinox

The relationship between number of particles used, number of iterations and median minimum distance of each point cloud to the average GPS locations for both time periods is illustrated in Fig. 3. Accuracy increases with increasing iterations and particles numbers, reaching an asymptote at around 60 iterations, and 300 and 800 particles during the solstice and equinox periods, respectively. Varying the shape parameter (*μ*) for the assumed twilight uncertainty distribution for both twilight events simultaneously from 1 to 4 and thereby increasing the possible range of error from ~8 min to ~2 h, while keeping the maximum probability at the input twilight timing, did not seem to affect the accuracy of the results for either time period (Additional file 6).

## Discussion

By comparing locations calculated from light and temperature data to concurrent GPS positions during two contrasting times of the year (close to the solstice and equinox), we demonstrated that our new method provides consistently high accuracy throughout the year, similar to the minimum uncertainty of the standard threshold method (i.e. during solstices at high latitudes; Table 4) [14, 30]. Tracks from two fast moving seabird species, black-browed and wandering albatrosses, could be reconstructed using this approach by incorporating additional environmental data (notably SST). In addition to providing positions around the equinox, this method provides an uncertainty associated with each computed position. This uncertainty could be used, for example, to build more realistic models of the measurement component of SSM for further behavioural analysis to account for the complex error structure of geolocations.

Our method for estimating locations from light-level data offers a simple, fast and intuitive approach accessible via the R package probGLS. This method is not Baysian or based on Kalman filter in contrast to the other statistically-advanced methods that are currently available (such as the R packages Trackit [25], SGAT/tripEstimation [26], and FlightR [27], Table 1) and we hope it will be less of a “black box” for many ecologists, with assumptions being more transparent at the expense of a mathematically rigorous framework. As with FlightR, our method generates a cloud of possible particles for each location, but uses probability sampling to construct a path rather than a particle filter. Further, the current implementation of probGLS takes about 30 min for a 1 year track (2000 particles, 100 iterations; Intel Core i7-3540 M 3 GHz, 16 GB RAM). This is to our knowledge faster than any SSM method (Table 1). With a run time per track of less than an hour it is feasible to run sensitivity analyses on input parameters (as in this study). Unlike the R packages based on SSM, probGLS cannot account for movement of the study animal between consecutive twilight events, which can reduce certainty in location estimation for certain taxa. However, it does not require the assumption or calibration of a constant solar angle throughout the year [20, 31], unlike the classical threshold method. The reason is that the added uncertainty around each twilight event as well as the range of solar angles accounts for different behaviour and levels of sensor shading around sunrise and sunset during the tracking period.

Twilight events for both albatross species computed by twilightCalc were not inspected manually for false or low-confidence transitions (reflecting interruptions to light records), and only outliers outside the defined boundary box (Table 3) were removed during processing. The range in accuracy, in particular for black-browed albatross data (Table 4), shows that the method was unable to correct twilight events which are far from the correct time (i.e. falsely assigned). These result in unreliable location clouds which the algorithm will attempt to fit into the movement path. However, most of these outliers were removed subsequently in the algorithm based on the assumed speed distribution, as well as land avoidance and SST weighting (steps 4 & 5). Accuracy could be improved if twilight events are either edited manually, filters such as loessFilter (GeoLight package) are applied, or the extent of the boundary box reduced before running the new method.

The number of particles needed for computation depends on the range of latitudes set in the parameter boundary.box (i.e. assumed latitudinal range during the equinox) as well as the longitudes defined through the parameters sunrise.sd and sunset.sd. We let latitude during the equinox vary by 90° (Table 3) as we did not expect the tracked individuals to cross the equator, whereas longitudinal uncertainty was assumed to vary over ~35 min to account for differences in shading due to behaviour and weather patterns (Table 3, Additional file 1). Based on Fig. 3, at least 800 particles are needed for stable results throughout the year. If the latitudinal uncertainty during the equinox is 180° (i.e. from pole to pole) the number of particles would need to be doubled. The minimum number of iterations needed for a consistent output was already reached at 60.

The median closest distance of each iterated location cloud to the mean GPS location of 19 and 17 km in the two time periods (Table 4) reflects the 0.25° spatial resolution of the satellite-derived SST dataset. Using a higher resolution SST dataset will likely increase the accuracy of this approach for this particular example. This illustrates that the selected weightings, as well as their resolution influence the accuracy and degree of uncertainty of a track. A high range of solar angles, a high uncertainty in twilight events and high assumed movement speed, combined with a lack of available environmental characteristics will lead to greater uncertainty and lower accuracy overall. Conversely, the accuracy of the method would increase if the range of solar angles as well as the twilight event uncertainty could be restricted based on previous knowledge (e.g. calibration periods).

We have demonstrated here that the algorithm achieves stable results with fast moving species in open landscapes (flying seabirds) and are optimistic that results would be comparable for animals inhabiting other habitats (e.g. terrestrial birds and diving organisms), especially if additional information to weight the computed particles is available. We already have preliminary indications that the algorithm performs well on diving species such as penguins. However, the suitability of the method for a wider range of species has to be confirmed in further studies.

## Conclusion

We presented an intuitive and time-efficient algorithm which makes it possible to analyse geolocator data from loggers of different types and manufacturers, deployed on any animal, throughout the year, including equinox periods (if sufficient additional information is available), in a consistent way, while acknowledging the limitations and uncertainties associated with light data. We do not claim that it is the most accurate method, but rather that it can be used widely and easily, regardless of whether the data were processed using outmoded software or new methods, without requiring a subjective step in determining or filtering locations.

## Additional files

Additional file 1: Twilight event uncertainty structure. (PDF 362 kb)
Additional file 2: Geographic median description. (PDF 205 kb)
Additional file 3: Recorded ground speed frequencies. (PDF 246 kb)
Additional file 4: R script for sensitivity analyses. (TXT 9 kb)
Additional file 5: Histograms of deviation of GLS computed locations to average GPS locations using two methods of light level location estimation. (PDF 257 kb)
Additional file 6: Sensitivity analysis for changing shape parameters determining the twilight event uncertainty. (PDF 308 kb)

## Abbreviations

GLS: global location sensor
GPS: global positioning system
SSM: state space model
SST: sea surface temperature
