# Supplementary material for: A probabilistic algorithm to process geolocation data
Source: Mov Ecol. 2016 Nov 18;4:26. doi: 10.1186/s40462-016-0091-8 (PMC5116194; doi:10.1186/s40462-016-0091-8)
Supplement: Additional file 1: — Twilight event uncertainty structure. (PDF 362 kb) [file 40462_2016_91_MOESM1_ESM.pdf]

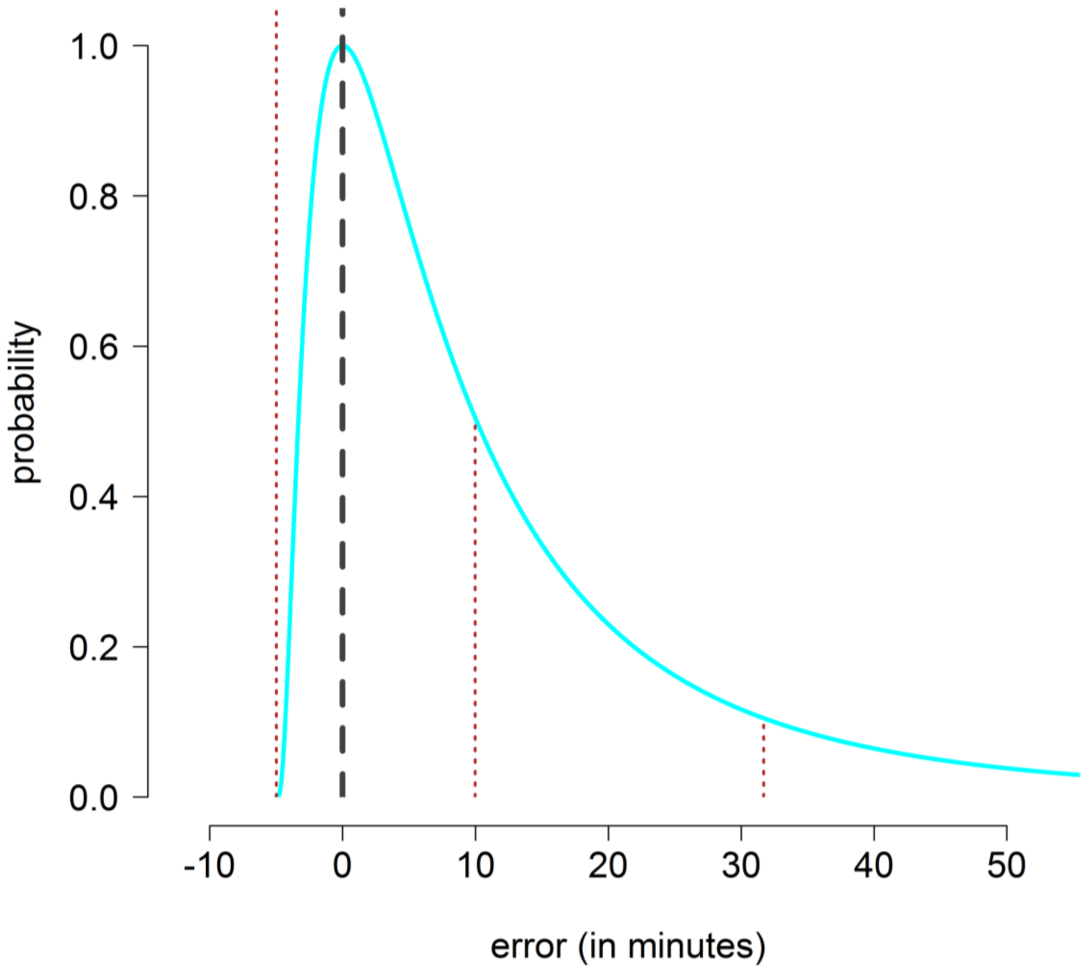

Figure A1. A twilight event uncertainty structure, assuming a log normal distribution, as determined by the function `twilight_error_estimation` (`probGLS` package). This distribution depicts the uncertainty structure around a sunrise and would be mirrored along the stripped line for a sunset. For this study the values of the "shape" ($log(\mu)$), "scale" ($log(\sigma^2)$) and "delay" parameters have been set to 2.49, 0.94 and 0, respectively. The uncertainty represents a maximum error before sunrise/ after sunset of 5 minutes, 50% probability error after sunrise/ before sunset of 10 minutes and 10% error after sunrise/ before sunset of 31.7 minutes. The parameters are chosen as they resemble the twilight error structure of open habitat species described in [1].

1. Lisovski S, Hewson CM, Klaassen RHG, Korner-Nievergelt F, Kristensen MW, Hahn S: **Geolocation by light: accuracy and precision affected by environmental factors.** *Methods in Ecology and Evolution* 2012, **3:**603-612.
