# Supplementary material for: A probabilistic algorithm to process geolocation data
Source: Mov Ecol. 2016 Nov 18;4:26. doi: 10.1186/s40462-016-0091-8 (PMC5116194; doi:10.1186/s40462-016-0091-8)
Supplement: Additional file 2: — Geographic median description. (PDF 205 kb) [file 40462_2016_91_MOESM2_ESM.pdf]

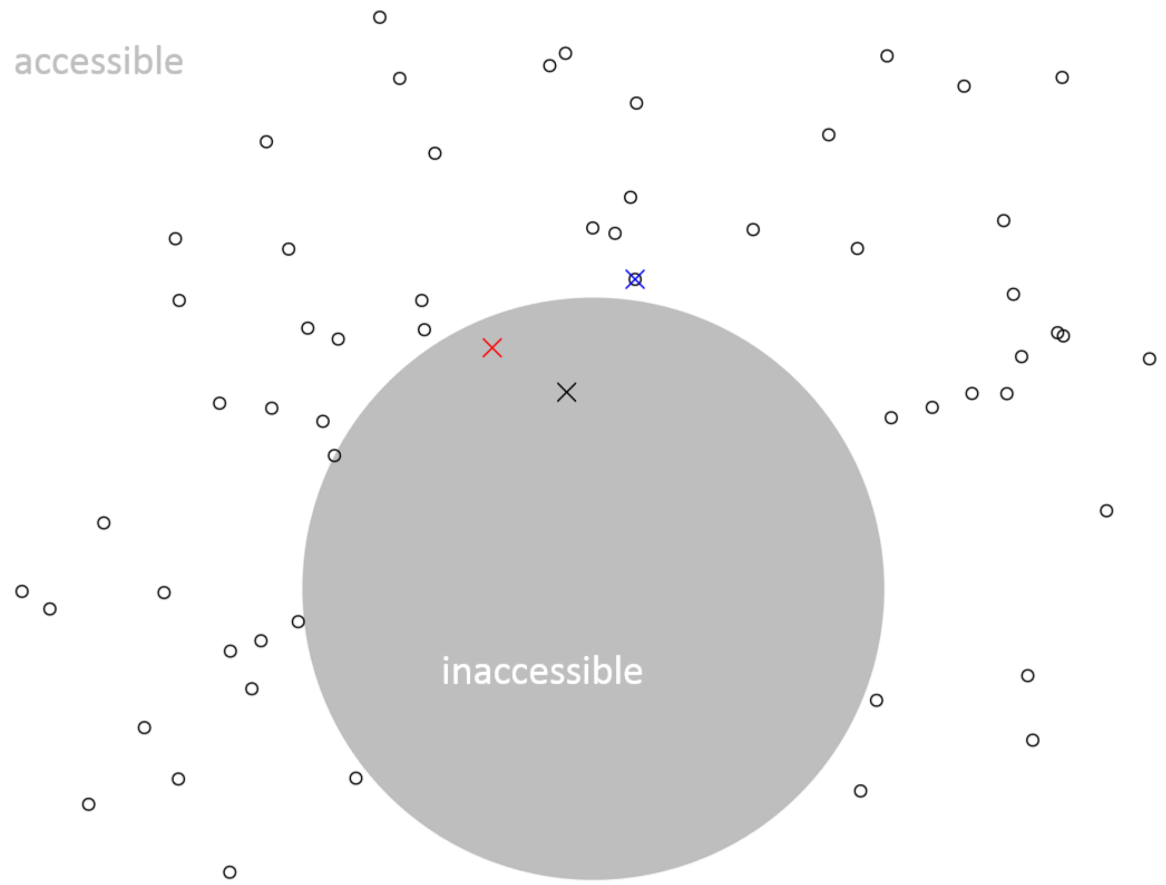

Figure A2. Example figure illustrating the difference between averaging latitude and longitude (black cross), taking the median of latitude and longitude (red cross) and calculating the minimum sum of all distances (geographic median) to all other locations (blue cross) around an inaccessible area (grey circle). Both averaging as well as the median results in placing the so calculated position inside the inaccessible area while the geographic median is placed outside this area as it represents one of the original locations.
