# Supplementary material for: A probabilistic algorithm to process geolocation data
Source: Mov Ecol. 2016 Nov 18;4:26. doi: 10.1186/s40462-016-0091-8 (PMC5116194; doi:10.1186/s40462-016-0091-8)
Supplement: Additional file 3: — Recorded ground speed frequencies. (PDF 246 kb) [file 40462_2016_91_MOESM3_ESM.pdf]

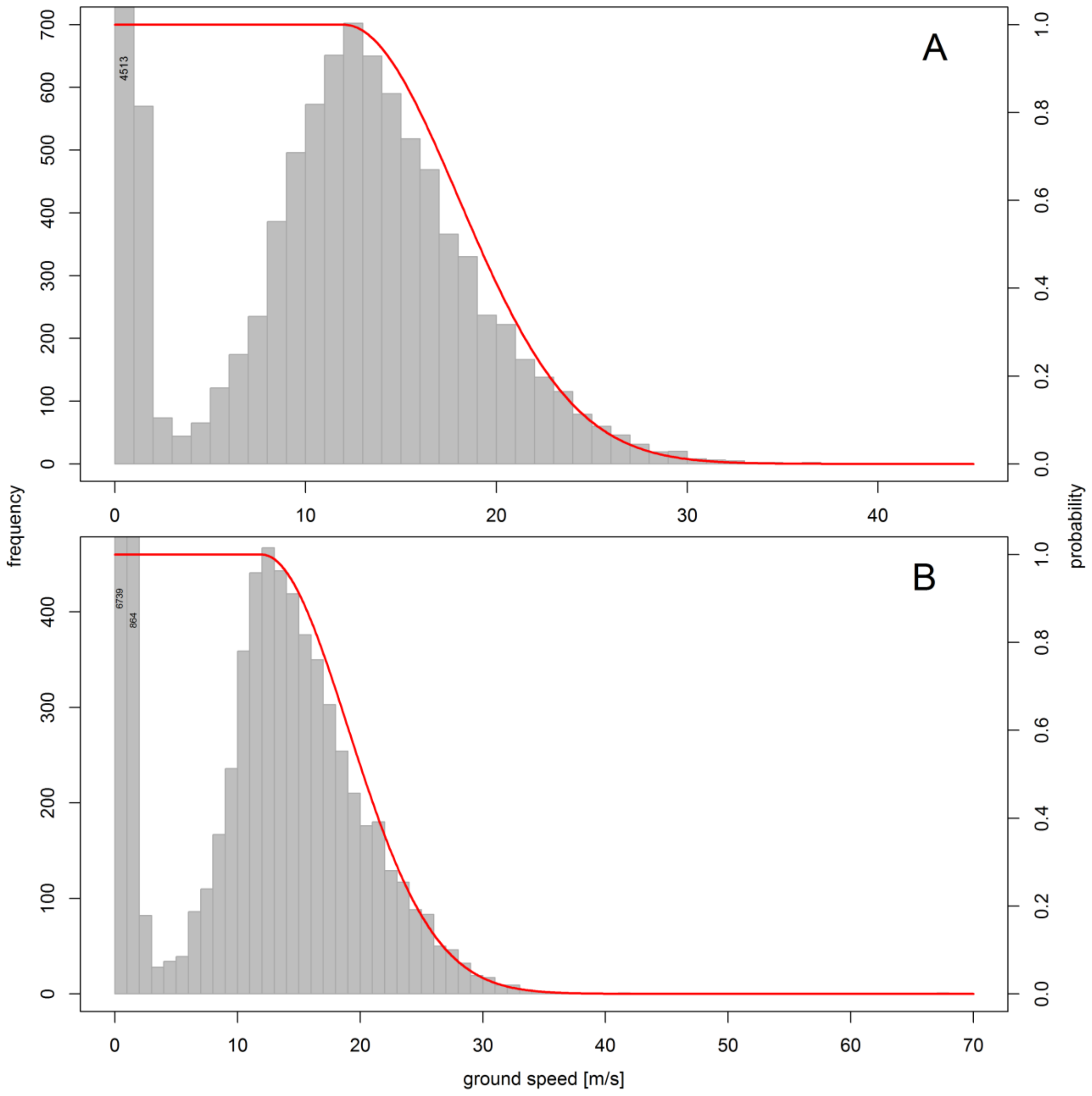

Figure A3. Recorded ground speed frequencies (in grey) and assumed ground speed probabilities (red line). Speed values used in the algorithm for black-browed albatross (A) as well as wandering albatross (B) are recorded in Table 2. Note that lower speeds are also assumed to have a high probability so as not to have to specify behaviour states as in state space models.
