# Supplementary material for: A probabilistic algorithm to process geolocation data
Source: Mov Ecol. 2016 Nov 18;4:26. doi: 10.1186/s40462-016-0091-8 (PMC5116194; doi:10.1186/s40462-016-0091-8)
Supplement: Additional file 5: — Histograms of deviation of GLS computed locations to average GPS locations using two methods of light level location estimation. (PDF 257 kb) [file 40462_2016_91_MOESM5_ESM.pdf]

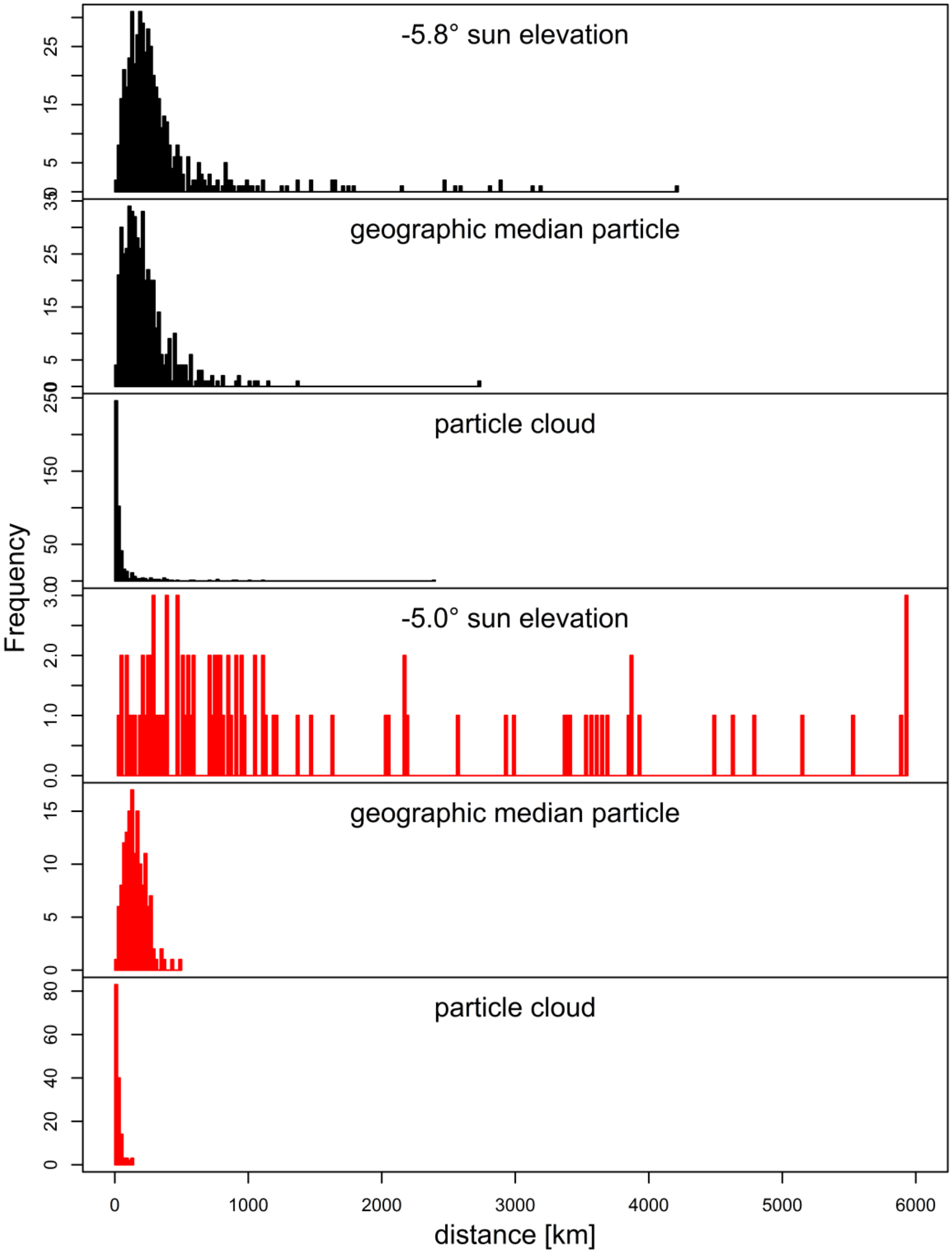
-5.8° sun elevation
geographic median particle
particle cloud
-5.0° sun elevation
geographic median particle
particle cloud
Frequency
distance [km]
0
1000
2000
3000
4000
5000
6000

Figure A5. Histograms of deviation of GLS computed locations to average GPS locations using two methods of light level location estimation. Geographic median particle refers to the calculated most probable movement track, and particle cloud refers to the minimum distance of the iterated particle cloud from the GPS location. Black-browed albatross data collected during the summer solstice in black (top three panels) and wandering albatross data collected during the fall equinox in red (bottom three panels).
