# Supplementary material for: A probabilistic algorithm to process geolocation data
Source: Mov Ecol. 2016 Nov 18;4:26. doi: 10.1186/s40462-016-0091-8 (PMC5116194; doi:10.1186/s40462-016-0091-8)
Supplement: Additional file 6: — Sensitivity analysis for changing shape parameters determining the twilight event uncertainty. (PDF 308 kb) [file 40462_2016_91_MOESM6_ESM.pdf]

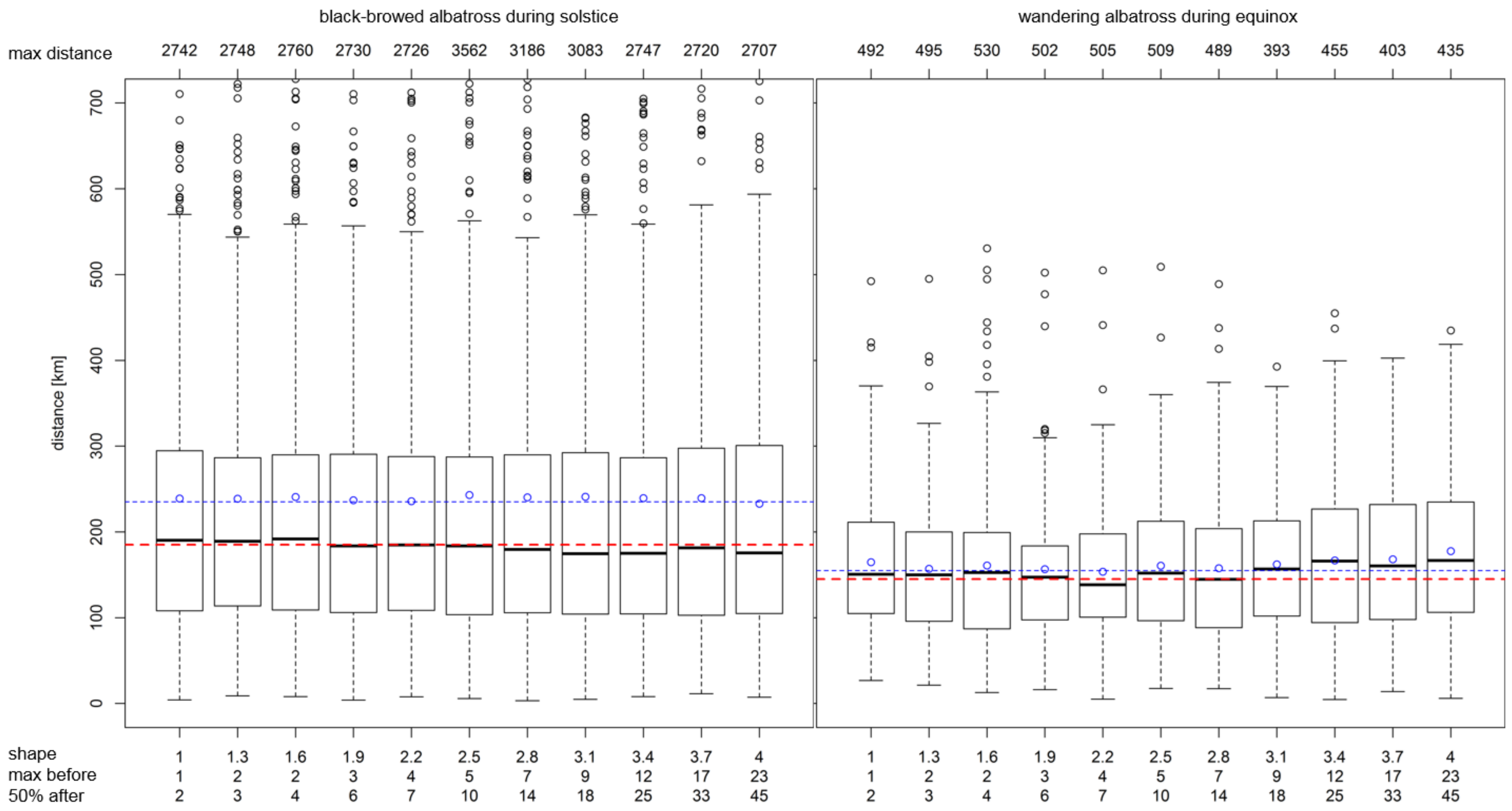
black-browed albatross during solstice
wandering albatross during equinox
max distance
2742 2748 2760 2730 2726 3562 3186 3083 2747 2720 2707
492 495 530 502 505 509 489 393 455 403 435
distance [km]
0 100 200 300 400 500 600 700
shape
max before
50% after
1 1.3 1.6 1.9 2.2 2.5 2.8 3.1 3.4 3.7 4
1 2 2 3 4 5 7 9 12 17 23
2 3 4 6 7 10 14 18 25 33 45

Figure A6. Sensitivity analysis for changing shape parameters determining the twilight event uncertainty. The y-axis denotes the distance to the average GPS position with the maximum distance displayed on top of each graph. Blue points – mean distance; black line – median distance; box – 25 % to 75 % inter quartile range; red and blue stripped lines – median and mean distance for a shape value of 2.49. Max before represents the number of minutes the uncertainty extends before a sunrise event/ after a sunset event, while 50% after represents the number of minutes after a sunrise event/ before a sunset event where the uncertainty reached a probability of 50% (for more details see Additional file 1).
